# Estimating the Effect of Recurrent Infectious Diseases on Nutritional Status: Sampling Frequency, Sample-size, and Bias

**DOI:** 10.3329/jhpn.v29i4.8447

**Published:** 2011-08

**Authors:** Wolf-Peter Schmidt, Bernd Genser, Stephen P. Luby, Zaid Chalabi

**Affiliations:** ^1^London School of Hygiene & Tropical Medicine, Keppel Street, London, UK; ^2^Instituto de Saúde Coletiva, Federal University of Bahia, Salvador, Brazil,; ^3^icddr,b, GPO Box 128, Dhaka 1000, Bangladesh

**Keywords:** Diarrhoea, Epidemiology, Infections, Measurement, Nutritional status

## Abstract

There is an ongoing interest in studying the effect of common recurrent infections and conditions, such as diarrhoea, respiratory infections, and fever, on the nutritional status of children at risk of malnutrition. Epidemiological studies exploring this association need to measure infections with sufficient accuracy to minimize bias in the effect estimates. A versatile model of common recurrent infections was used for exploring how many repeated measurements of disease are required to maximize the power and logistical efficiency of studies investigating the effect of infectious diseases on malnutrition without compromising the validity of the estimates. Depending on the prevalence and distribution of disease within a population, 15-30 repeat measurements per child over one year should be sufficient to provide unbiased estimates of the association between infections and nutritional status. Less-frequent measurements lead to a bias in the effect size towards zero, especially if disease is rare. In contrast, recall error can lead to exaggerated effect sizes. Recall periods of three days or shorter may be preferable compared to longer recall periods. The results showed that accurate estimation of the association between recurrent infections and nutritional status required closer follow-up of study participants than studies using recurrent infections as an outcome measure. The findings of the study provide guidance for choosing an appropriate sampling strategy to explore this association.

## INTRODUCTION

Nutritional status is an important risk factor for many infectious diseases in childhood (1-3) and for impairments in cognitive development and premature death (1). The effect of common recurrent infections and conditions, such as diarrhoea, respiratory infections, and fever, on gain in weight and growth of children is subject to an ongoing debate (4-6). To set the right public-health priorities to achieve sustained improvements in health and economic development in low-income settings, it is important to obtain a better understanding of the link between infectious diseases and nutrition (5).

Epidemiological studies exploring the effect of infections on nutritional status encounter a number of methodological challenges. In addition to confounding, another challenge is the choice of the surveillance strategy to measure the prevalence of infections which, in contrast to assessing the nutritional status of children, usually requires many repeated measurements. In an individual child, the presence or absence of infection is much more variable over time than, for example, the child's weight or height. It is not clear how precise the measurement of the prevalence of the infectious disease under study needs to be to estimate reliably the association between recurrent infections and nutritional status. The objective of this study was to identify the optimal balance between two contrasting barriers to obtaining valid estimates:

(a) If the measurement of the individual child's risk of infection is too imprecise (i.e. based on too few repeated measurements), the resulting estimate of the association between infection and nutrition will not only be imprecise but will also be biased towards null (7).

(b) If many repeat measurements are conducted, the information to be gained from further repeated measurements diminishes rapidly with the increasing number of measurements, i.e. after a certain number of measurements, the burden of disease of an individual child would be determined with sufficient accuracy and can hardly be improved by further visits (8). Also, many repeated household surveys require a large number of field workers who need to be recruited, trained, and supervised, often at high costs. Frequent visits to households participating in the study may compromise their willingness to be recruited, or to cooperate once the study is underway (8). There is evidence that reporting of disease by household members, usually the carer of the child, diminishes over time and at times causes a decline in measured disease prevalence which cannot be explained by changes in the age distribution and seasonal trends alone (9). Frequent visits may also change the healthcare-seeking and risk behaviour of households, thus influencing the outcome of interest (10). Parents may become more alert to symptoms and seek more medical care, or field workers may try to correct bad practices for professional and ethical reasons. For these different reasons, it is preferable to limit the number of repeated surveys to what is absolutely necessary.

Using a versatile model of common recurrent infections (11), we explored how many repeated measurements of disease are required to maximize the power and logistical efficiency of studies investigating the effect of infectious diseases on malnutrition without compromising the validity of the estimates.

## MATERIALS AND METHODS

### Description of model

The model is probabilistic. Its details are described elsewhere (11). The model has been used for evaluating the appropriateness of sampling strategies of common recurrent infections (8). In this study, it was used for investigating the sampling strategies required for exploring the effect of infectious diseases on malnutrition.

The basic model is summarized briefly as follows: The model simulates the daily variation of recurrent infections in individuals in a hypothetical population over one year. It is characterized by three components: incidence of episode, duration of episode, and a linear association between the incidence and the mean duration of episode, since individuals with more episodes tend to suffer from longer episodes (11). The incidence of episode and the duration of episode are modelled separately by Gamma distributions. Gamma distributions are useful to model highly skewed data, such as an individual's number of episodes of disease over a specified time period, where most individuals experience few episodes while a small number of high-risk individuals experience many episodes (11). All model parameters (i.e. those characterizing the probability distributions of incidence of episode and duration of episode and the positive correlation between incidence and duration) are estimated by least squares fitting of the model to observations of data from different field trials.

**Fig. 1.**
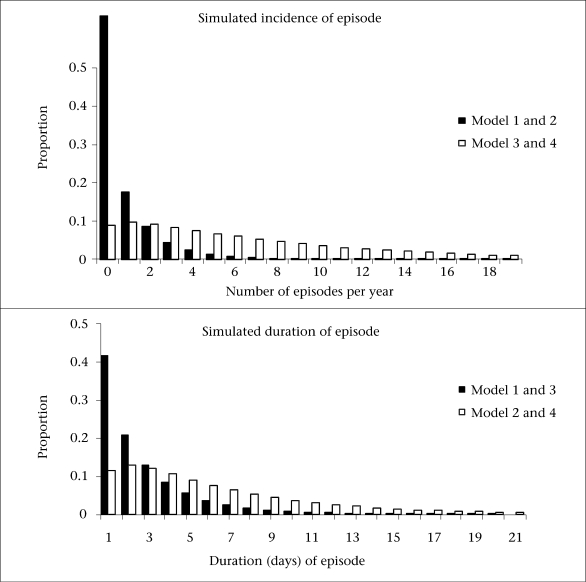
Assumed gamma distributions for incidence and duration of episode, reproduced from (8)

To circumvent the need for performing many simulations, four model scenarios were generated using permutations of parameter values to represent realistic and contrasting epidemiological scenarios observed in the field. Specifically, the scenarios and the model parameters were derived from longitudinal studies on diarrhoea, respiratory infections, and fever conducted in Brazil, Guatamala, Ghana, Thailand, Bangladesh, and other countries (8,11). The scenarios can be conceptualized by a two-by-two matrix in which the rows correspond to disease-incidence risk (low/high) and the columns correspond to duration of episode (short/long) Fig. 1 and Table 1).

The four scenarios are: (a) low incidence of disease and short duration of episode (LS), (b) low incidence of disease and long duration of episode (LL), (c) high incidence of disease and short duration of episode (HS), and high incidence of disease and long duration of episode (HL).

### Simulation of nutritional status

The effect of recurrent infections on nutritional status was simulated as a linear association between the proportion of time ill during 365 days of follow-up and the gain in weight of a child during that period. We used the proportion of time ill [subsequently termed ‘longitudinal prevalence’ (LP)] (12,13) as the exposure since this measure has been shown to be a better predictor of weight gain than the number of episodes (incidence) (12, 14) and has, therefore, been used in many studies on the effect of infections on nutritional status (4,14-18). For simplicity, we only considered a one way causal association between infection and weight gain, ignoring that nutritional status can have an effect on the risk of infection (2).

We used data from a large vitamin A trial in Ghana as parameters of weight gain over the simulated study period (19). Based on these data, we assumed that the difference in weight between the end and the beginning of the one-year observation period in children aged less than two years follows a normal distribution with a mean of 2 kg and a standard deviation of 0.8 kg. From the values of the weight gain for each child, we subtracted an amount that fully depended on the longitudinal prevalence of disease in that child over the simulated period of 365 days. Thus, we assumed that the association between disease and weight gain can be expressed by the following equation:

Weight gain (kg)=2.0-β*LP+ϵ [1]

where β is the regression coefficient of the linear association between disease and weight gain; LP is the longitudinal prevalence (=proportion of time ill) of an individual; and ϵ is the deviation of the simulated weight gain from the mean (i.e. epsilon is a normally-distributed variable of mean zero and standard deviation of 0.8).

For the slope parameter β we assumed that gain in weight decreases with every additional percentage point LP by 25 g, which corresponds approximately to published data based on field observations (12,14,18).

Importantly, the choice of the measure for nutritional status (alternatives would have been, for example, gain in height, or weight/height-for-age z-scores) did not influence the model output (see Discussion).

### Simulated surveillance strategies

In addition to simulating the daily time course of recurrent infections of a hypothetical population of individuals in the four contrasting scenarios described earlier, the model was also used for simulating surveillance visits at various intervals. At each visit, the model simulated the measurement of point prevalence by asking the hypothetical interviewee or their carer the question “on which of the previous 7 days did you have the disease” and taking into account the probability of recall error by the interviewee. The types of recall error and their details are given elsewhere (8). To summarize, the model assumed that if the disease was present at two days before the visit, it is always reported (i.e. with 100% probability) whereas if the disease was present earlier than two days, the probability of it being reported decreases the longer the lag period (23) (Table 2). The model was also used for simulating the measurment weekly period prevalence (i.e. “did you have the disease at any time during the last week”) which provides information on the number of weeks instead of days with the disease. This measure was used in many diarrhoea-intervention studies (24,25), especially in demographic health surveys.

**Table 1.** Four model scenarios with examples as an outcome

Incidence	Short duration of episode (α=0.8, β=2.7)	Long duration of episode (α=1.3,β=4.6)
Low incidence (α=0.6, β=2.1)	Model scenario 1 (LS)	Model scenario 2 (LL)
	Annual incidence: 0.9/person-year	Annual incidence: 0.9/person-year
	Mean duration of episode: 2.7 days	Mean duration of episode: 5.6 days
	Examples	Examples
	-Diarrhoea or fever in low-risk child population (20)	-ALRI in malnourished child populations (19,21)
		-Diarrhoea in an area with very heterogeneous risk (22)
High incidence (α=1.2, β=6.8)	Model scenario 3 (HS)	Model scenario 4 (HL)
	Annual incidence: 7.0/person-year	Annual incidence: 7.0/person-year
	Mean duration of episode: 2.7 days	Mean duration of episode: 5.6 days
	Examples	Examples
	-Diarrhoea or fever in high-risk child populations (2,21)	-Diarrhoea in very poor settings in undernourished children (19)
		-Mild ARI in high-risk population (19,21)

α and β values correspond to the parameters of the specified gamma distribution; reproduced from (8).

ALRI=Acute lower respiratory infection;

ARI=Acute respiratory infection;

HL=High incidence of disease and long duration of episode;

HS=High incidence of disease and short duration of episode;

LL=Low incidence of disease and long duration of episode;

LS=Low incidence of disease and short duration of episode

### Analysis

The longitudinal estimates of prevalence resulting from the different sampling strategies represent estimates of the ‘true’ proportion of time ill in a child, here defined as the proportion of time ill if all 365 days had been recorded with 100% accuracy. Since the simulated association between disease and weight gain depends fully on the ‘true’ longitudinal prevalence, we were able to explore how the ‘true’ association is estimated if the longitudinal prevalence estimates are based on fewer measurements, i.e. are less precise. We did this using linear regression, with the regression coefficient (i.e. the decrease in weight gain per additional percentage point of longitudinal prevalence) as the model output. We assumed a decreasing number of visits during 365 days of the study duration, i.e. 52 (=weekly visits), 40, 30, 20, 15, 12, 10, 8, 6, 4, 2, and finally a single visit. The model results were further used for estimating the required sample-size of a study using different sampling strategies. We used a relationship allowing the sample-size calculation for linear regression for sufficiently large sample-sizes, which we adapted from that published by Dupont and Plummer (26).

**Table 2.** Recall error

Day before surveillance visit	Probability of reporting disease
-1	1.0
-2	1.0
-3	0.74
-4	0.67
-5	0.67
-6	0.58
-7	0.58

Values based on (23)

n=(*u*+*v*)^2^/(βSD_LP_/SD_resid_)^2^ [2]

where *u* is the standard normal deviate value corresponding to the study power (0.84 for 80% power); *v* is the standard normal deviate value corresponding to the assumed significance level (1.96 for p=0.05); β is the expected value for the regression slope; SD_LP_ is the standard deviation of the dependent variable (in this case the longitudinal prevalence of disease); and SD_resid_ is the standard deviation of the residuals of the regression line. The simulations were done in the Stata software (version 9.0). All results were averaged over 500 runs which were found to be sufficient to achieve robust estimates.

## RESULTS

**Fig. 2.**
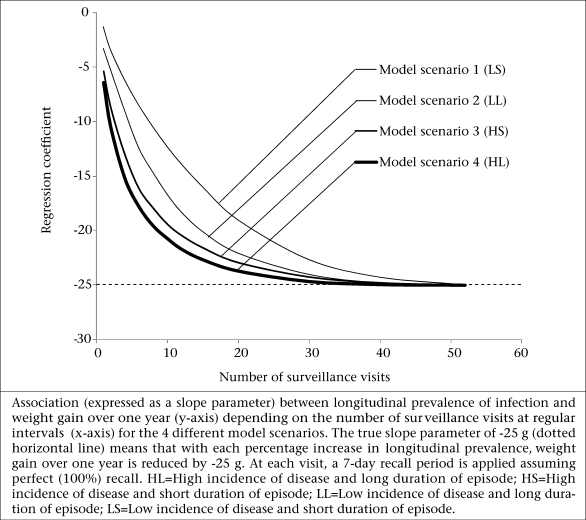
Association between number of visits and effect size

Figure 2 shows the association between the number of surveillance visits and the size of the estimate (the slope parameter – weight gain per year), assuming a seven-day recall period at each visit and *no* recall error. Therefore, 52 visits (once every week) with seven-day recall provide continuous disease records without gaps. The figure shows that if the frequency of surveillance decreases from 52 visits, the estimate is biased from the true value of −25 g (per percentage point of the LP) towards zero. The effect of bias is particularly pronounced for the low-prevalence model scenario 1 (LS).

**Fig. 3.**
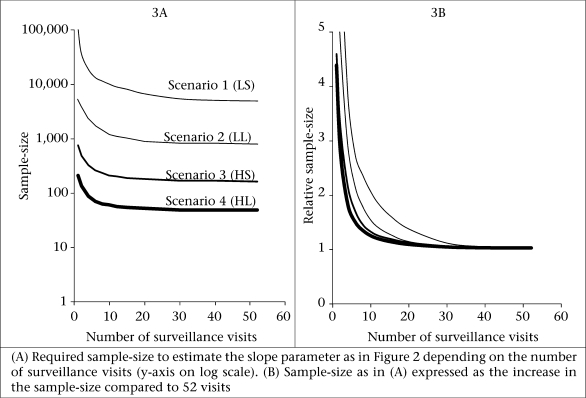
Association between number of visits and sample-size

Figure 3A shows the association between the number of visits and the required sample-size to estimate the slope parameter, using Equation [2] and assuming 80% power and p=0.05. The sample-size takes into account the bias in the estimate shown in Figure 2. The absolute sample-sizes are much higher for the low-prevalence scenario 1 and 2 (LS and LL) but the relative increase with the decreasing frequency of visit is also more pronounced in these scenarios. This is highlighted in Figure 3B which shows the relative increase in the sample-size with the sample-size for 52 visits as the reference.

**Fig. 4.**
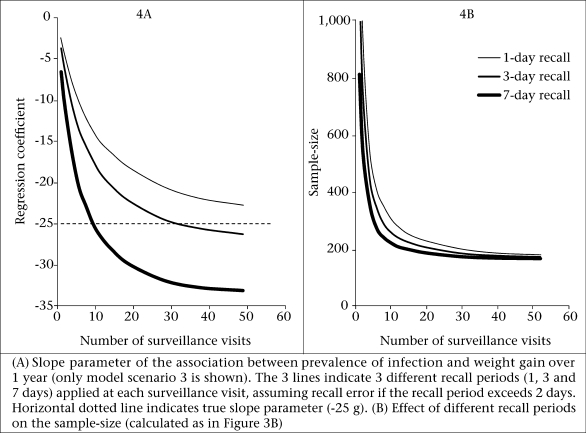
Effect of recall error on effect size and sample-size

We then introduced recall error into the models by assuming that the probability of an infection being reported decreases if the day is more than two days before the day of the simulated visit (Table 2). This meant that, on average, only 75% of days with infection were recorded as such. For illustration, the following calculations were done only for model scenario 3 (HS) (the same analysis conducted for the other models in principal resulted in similar findings). Figure 4A shows the effect recall error on the regression coefficient. For a recall period of seven days, recall error results in an exaggerated estimate of the regression coefficient biased from an expected value of 25 g to 32 g per percentage point LP (for frequent visits). For comparison purposes, Figure 4A also shows the effect of restricting the recall period to one day (where 100% recall was assumed) and three days (recall loss of 26% on day 3 before the visit, Table 2). A one-day recall period provides a small underestimation of the true regression coefficient (23 g instead of the true value of 25 g per percentage point LP). Using one-day recall period at 52 visits is not precise enough to provide a fully-unbiased estimate. In contrast, a three-day recall period resulted in a small overestimation from an expected value of 25-26 g per percentage point LP.

Figure 4B shows the effect of different recall periods (with recall error assumed as in Figure 4A) on the required sample-size of epidemiological studies. A seven-day recall period requires the smallest sample-size but the difference in sample-size to that of a three-day recall period is not very large if the number of visits exceeds 20.

**Fig. 5.**
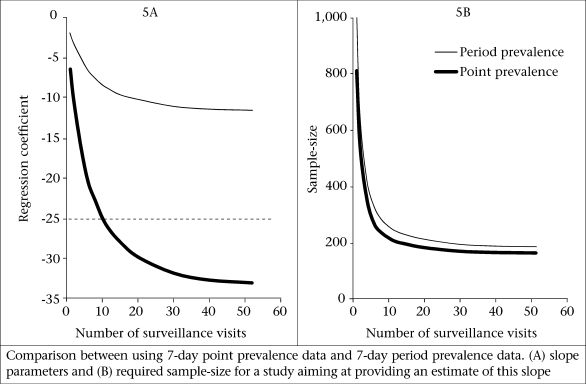
Point prevalence vs period prevalence to estimate the effect of infection on nutrition

Finally, we investigated the effect of using weekly period prevalence data rather than point prevalence data, assuming the same recall error (Table 2). Figure 5A shows the coefficients for weekly period prevalence data as a function of the number of visits (the coefficients for using seven-day point prevalence data as in Figure 4A are shown for comparison). The estimated regression coefficients for weekly period prevalence data are much closer to zero than the coefficients for point prevalence data because they measure the change in gain in weight per additional percentage point LP measured as the proportion of *weeks* with illness. Although period prevalence data are much less precise in recording the prevalence of infection than point prevalence data, the sample-sizes required for a study using period prevalence data are only slightly larger than for studies using seven-day point prevalence data (Fig. 5B).

## DISCUSSION

When deciding about the best sampling strategy for exploring the association between common infectious diseases and nutritional status, one needs to consider the effect of the sampling frequency and recall period on the size of the estimate and the required sample-size. Our analysis shows that random error in the measurement of the disease estimates (e.g. due to infrequent visits) can lead to estimates biased towards zero while recall error may inflate the size of the effect.

Broadly, our analysis suggests the following rules:

(a) To obtain estimates within 10% of the true association (which for most research questions may be acceptable), one has to conduct around 30 visits under scenario 1 (low incidence, short duration), 22 visits under scenario 2 (low incidence, long duration), 18 visits under scenario 3 (high incidence, short duration), and 15 visits under scenario 4 (high incidence, long duration).

(b) A three-day recall period as used in some previous studies (9,21,27) may be the best compromise between the risk of inflating the estimate due to recall error and the risk of biasing the estimates towards null due to imprecision. However, the final choice of the recall period may depend on the study setting and should follow pilot testing in the field.

(c) There appears to be little advantage of conducting very many visits (for example in excess of 25), as the gains in precision and minimization of bias are unlikely to outweigh the logistical effort and the potential for jeopardizing the willingness of participants to cooperate or influence reporting and risk behaviour.

In a previous publication, we investigated the effect of varying the sampling frequency on the sample-size of studies in which the longitudinal prevalence of infection is the outcome rather than (as in this paper) the exposure (8). We found that, in studies with a very low budget for carrying out surveillance visits, few visits at long intervals may be an inexpensive and efficient alternative to more intensive surveillance. If the longitudinal prevalence of infection is the outcome measure, the sampling frequency only affects the precision of the estimates such as risk ratio, not its size, regardless of how precisely disease is measured.

In contrast, if (as in this analysis) the prevalence of infection is the *exposure* variable, the sampling frequency not only influences the precision and power of a study but also the potential for bias (7), the magnitude of which we aimed at quantifying in this analysis. The analysis provides guidance on the minimum number of visits necessary to obtain valid estimates between recurrent infections and nutritional status. A recent article describing a randomized water-quality intervention trial found strong support for minimizing the number of repeat measurements (10). Participants who were followed up frequently reported less diarrhoea than those sampled at long intervals, possibly due to higher recall error and ‘reporting fatigue’. Our model predicts that, in this situation, recall error can lead to bias. Had in this study frequent sampling been used for measuring an association between diarrhoea and nutritional status, the resulting effect estimates probably would have been exaggerated.

However, regardless of whether the longitudinal prevalence of infection is the outcome or the exposure measure, fewer surveillance visits will always require a larger sample-size. In a separate paper, we estimated for each of the four model scenarios (Table 1). factors by which the required sample-size needs to be increased to achieve the same power as continuous sampling over one year (8). For example, if a researcher plans to conduct 20 visits instead of 52 over one year (assuming a seven-day recall period), the sample-size needs to be increased by 10% given model scenario 3 (HS) (8). We found these inflation factors to be identical to those applicable when calculating the sample-size of studies with the prevalence of infection as the exposure variable (Fig. 4B).

We found in this study that using weekly period prevalence data rather than point prevalence data only requires a slightly larger sample-size but the resulting regression coefficients are less intuitive as they describe the loss in weight gain per week in which infection occurred at any time. Also, as we have shown previously, weekly period prevalence data are unsuitable as a measure of disease if the effect of infections on nutritional status is largely due to the differences in the duration of illness but not due to the differences in incidence (8). This is because individuals who had diarrhoea at some point during the last seven days may have suffered from one or more episodes of different duration. The number of diarrhoea days in the last seven days in these individuals may be anything between one and seven but when period prevalence data are recorded they are all simply coded as ‘diseased at any time during the last 7 days’.

We chose gain in weight over one year as outcome as a relatively intuitive measure for researchers who are not experts in nutrition. In a sensitivity analysis, we tested a range of values for the mean and standard deviation of the nutritional status measure and the slope parameter of the association between infections and nutritional status. We found that the choice of these values did not affect the model results. This finding may at first appear counter-intuitive given the wide range of different outcome measures used in nutrition research, such as height-for-age, weight-for-height, or weight-for-age. Some of these measures, such as absolute height, commonly increase or remain constant whereas relative measures (e.g. z-scores) can increase or decrease. While the choice of different outcome measures and of the associated standard deviations has a profound impact on the absolute sample-size, the relative changes in the sample-size due to different sampling frequencies remain constant. It should be noted, however, that our study does not include sample-size considerations for repeated measurements of nutritional status or for interactions, e.g. between exposure (longitudinal prevalence of infection) and time. More complex analyses with additional assumptions will be needed for such purposes.

We limited the number of scenarios to just four covering a fairly wide range of epidemiological settings and conditions (11). As with any more complex model, the choice of these scenarios was to some extent arbitrary but the principal conclusion of these analyses was consistent across these contrasting scenarios.

In the models incorporating recall error (Fig. 4 and Fig. 5), our assumptions regarding recall probability of disease on a given day before the visit were based on published data (23). These, however, may overestimate recall error, since it is plausible that the higher prevalence of infection closer to the surveillance visit simply indicates that household members remember disease during the last seven days as having occurred more recently than was actually the case. We assumed that recall error occurred independent of the overall disease risk of an individual. In reality, it may be that recall error is more pronounced in those at a high risk of disease, which may increase the bias in the association between disease and weight gain demonstrated in Figure 4 even further.

### Conclusions

Our analysis confirms the risk of bias introduced by measurement error in the exposure variable as described in other fields of epidemiologic research (7). Our results could provide guidance for choosing an appropriate sampling strategy to explore the association between recurrent infections and nutritional status—or any other outcome variable of interest.

## ACKNOWLEDGEMENTS

The study was funded by the Wellcome Trust (Grant No. WT082569AIA). The authors thank Saul Morris, Mauricio L. Barreto, and Wim van der Hoek for providing data for the model. They thank Sandy Cairncross, Thomas Clasen, and Suzanne Filteau for helpful comments and advice.
